# Interactions Between Ferroptosis and Oxidative Stress in Ischemic Stroke

**DOI:** 10.3390/antiox13111329

**Published:** 2024-10-30

**Authors:** Daohang Liu, Sha Yang, Shuguang Yu

**Affiliations:** College of Acupuncture and Massage, Chengdu University of Traditional Chinese Medicine, Chengdu 610075, China; dorian.lieu@gmail.com

**Keywords:** Smyd-2, Nrf-2, ischemic stroke, ferroptosis, lipid peroxidation, hinge and latch, ferroptosis, PUFAs, subcellular organelle

## Abstract

Ischemic stroke is a devastating condition that occurs due to the interruption of blood flow to the brain, resulting in a range of cellular and molecular changes. In recent years, there has been growing interest in the role of ferroptosis, a newly identified form of regulated cell death, in ischemic stroke. Ferroptosis is driven by the accumulation of lipid peroxides and is characterized by the loss of membrane integrity. Additionally, oxidative stress, which refers to an imbalance between prooxidants and antioxidants, is a hallmark of ischemic stroke and significantly contributes to the pathogenesis of the disease. In this review, we explore the interactions between ferroptosis and oxidative stress in ischemic stroke. We examine the underlying mechanisms through which oxidative stress induces ferroptosis and how ferroptosis, in turn, exacerbates oxidative stress. Furthermore, we discuss potential therapeutic strategies that target both ferroptosis and oxidative stress in the treatment of ischemic stroke. Overall, this review highlights the complex interplay between ferroptosis and oxidative stress in ischemic stroke and underscores the need for further research to identify novel therapeutic targets for this condition.

## 1. Introduction

Stroke is a major health concern affecting middle-aged and elderly individuals globally, with approximately 80% of cases being ischemic in nature [1]. The prognosis for ischemic stroke remains poor, with a high mortality rate of 45%, due to its complex mechanisms, including mitochondrial dysfunction, dopamine toxicity, oxidative stress, and metabolic disturbances [2]. Reactive oxygen species (ROS) play a crucial role in this process, being generated during cerebral ischemia/reperfusion (CIR) [3,4]. During reperfusion, increased blood flow leads to heightened ROS formation, causing secondary damage to the cerebrovascular system and neural networks [5,6,7,8]. These ROS can irreversibly damage DNA and other macromolecules, exacerbating CIR injury.

The primary antioxidant systems in the circulation, including enzymatic antioxidants like superoxide dismutase (SOD), catalase (CAT), paraoxonase-1 (PON-1), and glutathione peroxidase (GPx), work to neutralize reactive oxygen species, while non-enzymatic antioxidants such as glutathione, vitamins C and E, and uric acid play complementary roles by scavenging free radicals and protecting cellular components from oxidative damage. Together, these antioxidants form a complex defense network that maintains redox balance in the bloodstream, which is crucial in preventing oxidative stress-related diseases and preserving cellular health. This antioxidant system is particularly relevant in the context of ferroptosis, a type of programmed cell death driven by iron-dependent lipid ROS accumulation, which is implicated in various nervous system diseases, especially in stroke [9,10,11,12]. Characterized by distinct cellular and mitochondrial changes, ferroptosis differs significantly from other forms of cell death like apoptosis and necrosis, highlighting the need for robust antioxidant defenses to mitigate its damaging effects [13]. Myeloperoxidase, as an inducer of lipid oxidation, further complicates this balance by promoting lipid peroxidation and contributing to oxidative stress. This process is closely linked to glutathione depletion, which lowers the activity of glutathione peroxidase 4 (GPX4), leading to reduced cellular oxidation resistance and increased ROS production [11,14,15]. Acyl-CoA synthetase long-chain family member 4 (ACSL4) plays a key role in this process by converting polyunsaturated fatty acids into fatty acyl-CoA esters, which accelerate lipid peroxidation and damage cellular membranes [16,17]. Typically, lipid peroxidation damages the lipid bilayer by accelerating lipid membrane oxidation and decreasing membrane fluidity in various organelles, resulting in cytomembrane collapse, mitophagy, and endoplasmic reticulum (ER) stress [18,19,20]. NADPH, a critical molecule in cellular metabolism, has a dual role in ferroptosis in that it both supports the antioxidant glutathione system and contributes to lipid peroxidation [21,22]. Ferroptosis suppressor protein-1 (FSP-1) acts as a defense mechanism, reducing coenzyme Q to prevent lipid peroxidation [23]. Additionally, iron chelators and antioxidants like ferrostatin-1 and vitamin E have been shown to mitigate ferroptosis-related neuronal damage in stroke models [24,25].

Intravenous thrombolysis (IVT) with tissue plasminogen activator (tPA) is currently the only FDA-approved treatment for ischemic stroke [26,27]. However, its limitations, including a narrow treatment window and the risk of hemorrhage, reduce its effectiveness [28]. Recent research suggests that ferroptosis inhibitors might offer a new therapeutic avenue, highlighting the need for targeted strategies that address both ferroptosis and oxidative stress. Understanding the relationship between ferroptosis-induced ROS and cellular organelles could pave the way for innovative treatments for ischemic stroke.

## 2. Iron and Ischemic Stroke: Playing with Fire Will Get You Burned

Macrophages play a critical role in the clearance of senescent red blood cells by engulfing them and breaking down hemoglobin to recycle iron. Hmox-1, a critical rate-limiting enzyme in heme catabolism, decomposes heme into three by-products, carbon monoxide, Fe^2+^, and biliverdin, releasing free Fe^2+^ into the body [29]. Most of the iron found in blood is stored in the form of ferric iron (Fe^3+^) [30]. Fe^3+^ in the blood can bind to transferrin receptor 1 (TfR-1) on the cell membrane to enter cells via stable endosomes. As early as 2011, Rathnasamy et al. found that hypoxia-induced periventricular white matter (PWM) injury leads to massive intracellular iron accumulation in amoeboid microglial cells (AMCs), as evidenced by Perls’ iron staining and the elevated protein levels of IRP-1, IRP-2, and TfR [31]. This accumulation is implicated in secondary injury post-stroke, highlighting the importance of iron homeostasis in neuroprotection and recovery. Meanwhile, Fe^3+^ can be reduced to ferrous iron (Fe^2+^) by the metal reductase STEAP-3 and is subsequently transported from the nucleus to the cytosol for storage by divalent metal transporter-1 (DMT-1, also known as SLC-11A2) [32]. Maintaining extracellular iron homeostasis is essential to mitigate the deleterious effects of iron overload. The role of ferritin and ferroportin (FPN) in oxidizing excess Fe^2+^ and facilitating its export as Fe^3+^ further emphasizes the interplay between iron metabolism and neuronal health [32]. Brown et al. recently reported that Prominin-2 (PROM-2), a transmembrane glycoprotein, promotes the formation of ferritin-containing multivesicular bodies (MVBs) and exosomes that transport iron out of cells, thereby inhibiting ferroptosis [33,34].

The Poly-(rC)-binding protein (PCBP) family proteins and nuclear receptor coactivator 4 (NCOA-4) have also been shown to be critical for iron release [35]. PCBP-1 is a multifunctional protein that acts as a cytoplasmic iron chaperone, binding and transferring iron to receptor proteins in mammalian cells along with its siblings PCBP-2 and heterogeneous nuclear ribonucleoprotein K (HNRNPK) [36,37,38]. Ishii et al. confirmed that the encoded protein PCBP-1 plays another role in translationally controlling 15-lipoxygenase mRNA, thereby causing severe neuronal oxidative stress [35]. Mori et al. demonstrated that the abnormal RNA metabolism triggered by PCBP-1 can form intranuclear inclusions characterized by eosinophilic transparent inclusion bodies in Huntington’s disease [36]. Additionally, PCBP-2 has been shown to fit into a sequence-specific alpha-globin mRNP complex, which can become part of TDP-43-positive cytoplasmic inclusions, destabilizing mitochondrial and ribosomal alpha-globin mRNA in neurons, and further disrupting the function of the mitochondrial electron transport chain (ETC) in patients with frontotemporal lobar degeneration (FTLD) and amyotrophic lateral sclerosis (ALS) [38]. NCOA-4, an equally important transcription coregulator, is known to mediate ferritin autophagy as a cargo receptor [39,40]. Evidence suggests that NCOA-4 can increase neuronal susceptibility to ferroptosis by binding to its heavy chain and transporting ferritin to the lysosomal machinery—the autophagosome—where it is degraded and free iron is released [39,41,42].

In brief, from the onset of cerebral ischemic stroke, the severe injury of the brain tissue, the breakdown of the blood–brain barrier (BBB), the cascade of inflammation, cytotoxic edema, and increased cerebral capillary permeability together lead to a large quantity of iron rushing from the blood into the cerebral parenchyma [43]. Thus, these findings have outlined the whole process of systemic iron circulation in stroke and some degenerative diseases, lifting the curtain on the mysterious molecular mechanisms that iron overload is involved in during the pathogenesis of most neurological diseases.

## 3. GPX4 Raises the “Iron Curtain” for Ferroptosis

GPX4, a crucial phospholipid hydroperoxidase, plays a key role in cellular defense by inhibiting the formation of free radicals and lipid peroxidation [23]. Using RNA interference mediated by an siRNA-expressing vector targeting GPX4 in HT-1080 cells, Yang et al. found that cells with low GPX4 expression are more sensitive to ferroptosis. Additionally, other compounds, such as DPI7 (Diphenyleneiodonium-7) and DPI10 (Diphenyleneiodonium-10), can directly inhibit GPX4 activity, while the upregulation of GPX4 expression results in ferroptosis resistance [44]. Moreover, both RSL3 and Erastin significantly promote ferroptosis by inducing the activation of lipid ROS [45]. Erastin blocks system XC− to decrease cystine uptake and lower glutathione levels, while RSL3 targets GPX4 to induce ferroptosis without affecting overall glutathione levels [46].

Converging research suggests that glutathione (GSH) is a crucial endogenous substrate of GPX4. The consumption of this cofactor can lead to the inactivation of phospholipid peroxidase [47]. Simultaneously, while GPX4 converts the reduced GSH to oxidized glutathione (GSSG), it also starts to reduce toxic lipid hydroperoxides (L-OOH) to their corresponding non-toxic alcohols (L-OH) or free hydrogen peroxide to water, which are considered the proximate executioners of ferroptosis [48]. This catalytic reaction occurs at the selenocysteine (Sec) within the catalytic center of GPX4 [49]. Moreover, cysteine is the essential limiting amino acid for intracellular GSH synthesis, most of which is captured through the cystine/glutamate reverse transporter (Xc^−^), so cysteine starvation gives rise to GSH depletion and all of its associated ferroptosis [50,51]. Interestingly, selenoproteins containing the 21st amino acid, selenocysteine, are rare. Selenocysteine is similar to cysteine but differs due to a substitution of selenium for sulfur. Most known selenoproteins also exist as cysteine-containing homologs [52]. Selenocysteine is one of the amino acids in the active center of GPX4, and its biosynthesis relies heavily on a co-translational integration mechanism. Selenocysteine depletion can result in embryonic death in mice, whereas cysteine knockout mice develop spontaneous seizures and almost always experience pre-weaning mortality [53].

**Figure 1:**
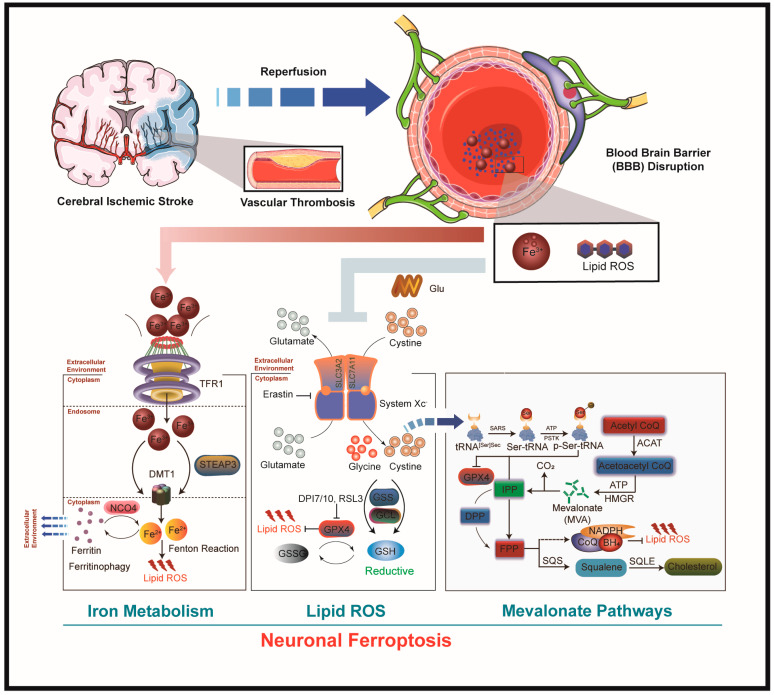
The blood–brain barrier is severely damaged during ischemic stroke. (1) After the resupply of blood and oxygen, ferrous ions are transported into the neuron nucleus with the assistance of membrane channel proteins such as TFR1, Steap3, and DMT1, where they are reduced to ferric ions. Then, the membrane is oxidized by hydroxyl radical · OH in the Fenton reaction in the presence of ferric ions. (2) Cerebral ischemia/reperfusion inhibits the activity of SLC7A11, reduces the transportation of cystine in neurons, and then reduces the synthesis of GSH. GPX4 could not reduce a large amount of lipid peroxides without sufficient substrate GSH, causing neuronal ferroptosis. (3) MVA pathways can act on GPX4 by regulating the maturation of selenocysteine tRNA, causing neuronal ferroptosis. Depletion of SQS competitively blocks neuronal ferroptosis.

In addition, mevalonate pathways (MVA or sometimes MEV) cause ferroptosis in the neurons of rodents and humans by weakening GPX4 activity [54]. A crucial component of MVA pathways is that selenocysteine can only be transported and inserted into GPX4 with the assistance of selenocysteine tRNA. Isopentenyl transferase, which transfers the isopentenyl group from isopentenyl pyrophosphate (IPP) to the selenocysteine tRNA precursor, is required for the maturation of selenocysteine tRNA [55]. In the zebrafish brain, statins are involved in ferroptosis as they inhibit HMG-CoA reductase, which is upstream of IPP [56]. Thus, GPX4, as a pivot regulator that protects neurons from ferroptosis by suppressing lipid peroxides, can be incapacitated by GSH consumption and selenocysteine. Together, these functions highlight the necessity of cysteine for GSH synthesis and the unique incorporation of selenocysteine into GPX4, indicating that disturbances in either amino acid could lead to enhanced neuronal ferroptotic susceptibility (summarized in Figure 1).

## 4. PUFAs: All About Their Past, Present, and Reincarnations

**Figure 2:**
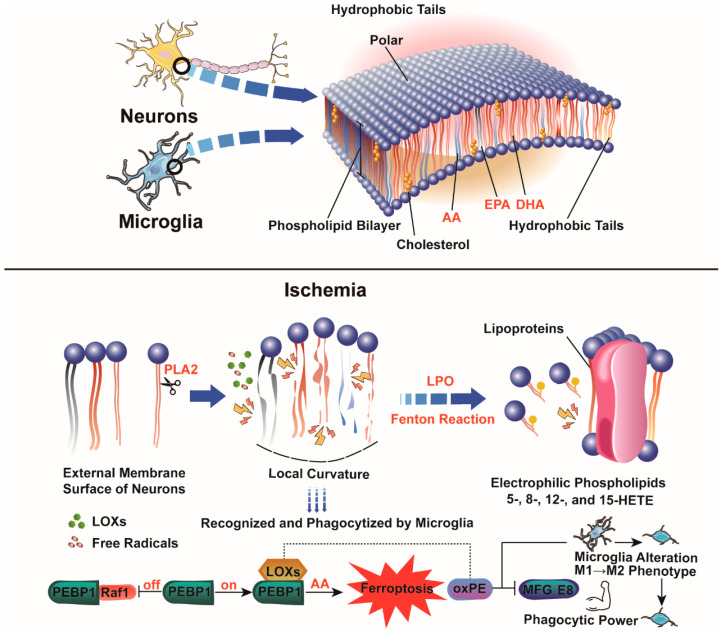
Schema illustrating the composition of the lipid membrane of neurons and microglia, which is susceptible to being oxidized by free radicals and phagocytized by microglia during ischemia; PEBP-1 dissociates from Raf-1 to interact with LOXs, which enlarges the phagocytic power of microglia toward neurons when cerebral infarction disrupts oxygen homeostasis.

Dried cod and herring are rich in protein, fatty acids with a linoleic acid base, Vitamin E, and glutathione. These nutrients are prominent in the diets of Eskimos, who rely on their excellent eyesight and cognitive abilities for hunting and fishing in their harsh environment. PUFAs, characterized by having more than one double bond in their backbone, are essential nutrients. They are classified into eicosapentaenoic acid (EPA) and docosahexaenoic acid (DHA) [36]. Additionally, PUFAs can be categorized into omega-3 and omega-6 PUFAs based on the position of the first methylene-interrupted double bond. Omega-3 PUFAs, associated with fairly sustainable effects on membrane integrity in contrast to the pro-inflammatory effect of omega-6 on account of the different downstream lipid metabolites, have been inundated by media attention and have aroused increasing interest regarding health promotion [57,58,59] (summarized in Figure 2).

Among omega-3 PUFAs, EPA and DHA are two key forms with significant bioactive properties in the body [60,61]. Their siblings, omega-6 PUFAs, are characterized by arachidonic acid (AA), which is essential in the development of the human brain and optic nerves [62]. However, the detailed mechanisms by which PUFAs are transported to the brain and influence cognitive function remain only partially understood. Research suggests that adequate dietary intake of omega-3 PUFAs can reverse cognitive decline and reduce the risks of neurologic disorders in animal models and humans [61,63,64]. Various nutrients, including omega-3 and omega-6 PUFAs in phospholipid-conjugated and unesterified forms, are routinely permeable to the BBB through simple non-saturable diffusion to maintain brain function due to the limited synthesis ability of omega-3 PUFAs from fatty acid precursors in the brain [65]. The amount of DHA absorbed in the brain through whole emulsion particle uptake is relatively low. Increased levels of plasma LPC-DHA and non-esterified DHA (NE-DHA) indicate that other transporters, like ApoE4 protein, can transport DHA within the central nervous system to maintain brain lipid homeostasis [66]. In contrast, EPA not only promotes the expression of neurotrophins, such as GDNF, NGF, and BDNF, in microglia, thereby exerting neuroprotective functions, but also reduces neuronal death in the hippocampus through the BCL-2 pathway and BDNF-TrkB signaling, compared to DHA [67]. Microglia are the primary synaptic phagocytes responsible for controlling synapse numbers in the brain. These highly motile cells move precisely toward damaged sites caused by ischemic, excitotoxic, and neurodegenerative insults, where they phagocytose neuronal debris and sculpt postnatal neural circuits, thereby providing constant surveillance and scavenging for neurodevelopment [68,69,70]. Genomic and proteomic tools have demonstrated that the modification of microglia fatty acid composition disturbs microglia homeostasis and crimps microglia phagocytic activity to synapses, further leading to the weakening or loss of connections in the hippocampus [71].

PUFAs dominate in neuronal survival, neurogenesis, neurotransmission, synapse formation, and neurite outgrowth. They maintain the fluidity of neuronal lipid membranes, which is essential for proper membrane function. PUFAs achieve this by contributing to a flexible, water-repellent membrane structure. Conversely, elevated membrane cholesterol levels resulting from a deficiency in PUFAs are associated with reduced membrane fluidity [72]. Notably, approximately 5% of the body’s energy is dedicated to repairing damaged lipids in the brain [73]. Shi et al. indicated that polyunsaturated fatty acids, as opposed to saturated or monounsaturated fatty acids, enhance basal neurotransmission and synaptic plasticity while normalizing synaptic structure in postsynaptic hippocampal CA1 neurons [74]. Vetrivel et al. observed that rats who received omega-3 fatty acids exhibited increased levels of Brain-Derived Neurotrophic Factor (BDNF), which helped counteract brain injury [75]. Senescent animals with lower-than-normal levels of n-3 polyunsaturated fatty acids show impaired function in glutamatergic synapses and exhibit a 30% reduction in glutamate uptake in astroglia from the CA1 region of the hippocampus [76,77,78]. However, a deficiency in maternal dietary n-3 PUFAs reduces hippocampal neuronal synaptic plasticity by significantly decreasing the expression of postsynaptic scaffold proteins PSD-95 and cofilin. This deficiency also promotes the production of pro-inflammatory mediators, which are internationally recognized as “pseudo-inflammation” or “sterile inflammation”, by shifting the offspring’s microglial phenotype from a defense-oriented M2 to a more inflammatory M1 phenotype [79,80,81]. Low n-3 PUFA levels are also associated with changes in dendritic arborization and slower cognitive processing speeds in the anterior cingulate cortex (ACC) and midbrain circuits, which are involved in balancing sensory and motor coordination in both primates and rodents [82,83,84]. Thus, PUFA deficiency is linked to a spectrum of neurodevelopmental issues and cognitive decline.

In ischemic stroke and similar traumatic events, interrupted cerebral blood flow limits glucose and oxygen delivery, increasing extracellular glutamate levels. This triggers neurotoxic activity and accelerates neuronal death. Hypoglycemic and hypoxic neurons may resist ferroptosis by upregulating glutamate oxaloacetate transaminase (GOT), which reduces glutamate toxicity and supports the Tricarboxylic Acid Cycle (TCA) to sustain neuronal viability [85]. This aligns with the idea that metabolic dysfunction causes energy failure and ROS buildup, leading to cell death through disruptions in cell membrane ion concentrations [85]. Interestingly, while glucose deprivation is often thought to inhibit ferroptosis, this effect heavily depends on AMPK activity. Activated AMPK can block PUFA synthesis during low-glucose conditions, protecting cells from ferroptosis, making it a potential therapeutic target for ischemic stroke [86,87,88,89,90,91]. This approach is more practical, as activating the energy stress-inducible survival program can protect cells from ischemia–reperfusion injury. On a broader level, this protective mechanism may represent the body’s first line of defense against organ damage caused by energy depletion. Therefore, PUFAs are associated with the etiology of various neurodevelopmental disorders and impairments across the lifespan, from birth to old age.

## 5. The Apostle Paul of Ferroptosis: For What Irresistible Reasons Have These Moderate Neurogenic PUFAs Been Metamorphosed into Picky Lipid Peroxides?

Polyunsaturated fatty acid-containing phospholipids (PUFA-PLs) gain membrane bending rigidity between the lipid bilayer leaflets, which is energy-efficient when deformation occurs [92]. These membranes are prone to oxidation during free radical production, leading to PUFA ester “stub” extension and “whisker” formation with high local curvature. This conformational change in the fatty acyl chain upon oxidation exposes the oxidized fatty acid to phagocyte recognition [93]. This vulnerability renders neurons with higher PUFA-PL levels more susceptible to lipoxygenases (LOXs) and free radicals than most cells. While lipid peroxidation (LPO) accompanies nearly all programmed cell death (PCD), cardiolipins (CLs) and phosphatidylethanolamines (PEs) show a distinct LPO preference during ferroptosis, confirmed by high-resolution protocols like liquid chromatography–mass spectrometry (LC-MS) and mass spectrometry imaging (MSI) [94,95].

AAs are a prominent type of cellular membrane PUFA-PL involved in neural connections, essential for the central nervous system. Free fatty acids, including AAs, rise in number following cerebral ischemia and can be metabolized into pro-resolving mediators like 5-, 8-, 12-, and 15-HETE through the LOX/PEBP-1 complex [96]. Under normoxic conditions, PEBP-1 binds to Raf-1 but dissociates to interact with 15-LOX during oxygen disruption [97]. LOXs’ catalytic center, with its nonheme-iron complex, serves a function in redox equilibrium, catalyzing lipid radical formation upon activation of Fe^3+^-OH. Non-enzymatic LPO driven by ferrous iron more efficiently disrupts membrane lipids, producing oxidatively truncated phospholipids that alter membrane lipoproteins (LSPs). Redox lipidomics in ferroptosis highlights specific PUFA-PLs’ susceptibility to LPO. The LPO cycle involves nonheme iron transferring valence electrons to form lipid hydroperoxide [98]. In brief, LPO is triggered by alkoxyl radicals created by ferrous irons from the hydroperoxide derivatives of lipids (LOOH), which are the inevitable downsides of aerobic metabolism. Once a specified threshold is exceeded, ferroptosis occurs.

Among the lipoxygenases (LOXs) involved in ischemic brain injuries, upregulation of 12/15-LOX contributes to neuronal death in the penumbra and peri-infarct areas. Specifically, 12/15-LOX acts as an endogenous activator of nuclear translocation and peroxisome proliferator-activated receptor-γ (PPAR-γ), a neuroprotective factor in cerebral ischemic reperfusion [99]. Pallast et al. reported that 12/15-LOX aggregates sequentially in the neuronal perinuclear compartment, leading to the leakage of ER-resident proteins during glutamate challenge and aggravating the transient focal ischemic insult [100]. The 12/15-LOX/12-HETE pathway enhances microglial phagocytic activity in periventricular leukomalacia (PVL), which is similar to ischemic stroke, causing necrosis in the periventricular white matter [101]. Also, 12/15-LOX is a target for addressing hemorrhagic transformation linked to t-PA treatment post-ischemia, which affects BBB integrity and vasogenic edema [102]. Cellular membrane phospholipids consist of phosphatidylethanolamine (PE), phosphatidylcholine (PC), phosphatidylserine (PS), phosphatidylinositol (PI), and sphingomyelin (SM). Additionally, 12/15-LOX preferentially integrates into membranes with PEBP-1, targeting PE for oxygenation. It modifies the sn2-15-HpETE-PE complex (oxPE), which binds proteins like MFG-E8 [103]. MFG-E8 participates in the process of microglia phagocytizing viable neurons. However, it can also inhibit phagocytosis in inflammatory macrophages and promote a shift to the microglia M2 phenotype when bound to oxPE on the plasma membrane of microglia, which serves as a key antigen-recognized signal for phagocytic microglia [104,105,106]. Therefore, it can be inferred that 12/15-LOX-induced oxPE species are documented in the plasma membranes of resident microglia as a required mechanism for neuron-phagocytic function, partially dependent on the strong binding of oxPE and MFG-E8. Hence, during focal cerebral ischemia injury, the ferroptosis-induced production of microglial hydroperoxy (-OOH) lipids or oxPE probably enlarges phagocytic power, which treacherously may not be for the microglia but for the neighboring neurons (summarized in Figure 2).

Phospholipase A2 (PLA-2) family members are crucial for hydrolyzing peroxidized phospholipids, particularly for removing arachidonic acid (AA) and docosahexaenoic acid (DHA) from membrane phospholipids, which is key in ferroptosis resolution [107]. PLA-2s include secretory PLA-2 (sPLA-2), calcium-independent PLA-2 (iPLA-2), and cytosolic PLA-2 (cPLA-2). Both cPLA-2α and iPLA-2β are particularly significant in neurological diseases linked to ferroptotic phospholipid remodeling [108,109]. iPLA-2β is thought to mainly process fatty acid de-acylation/re-acylation reactions. The yields of these reactions are the selective major resources for the release of DHA that is highly concentrated in the neuronal plasma membrane [110]. Conversely, cPLA-2 is linked to inflammatory reactions, primarily releasing AAs via extracellular signal-regulated kinase (ERK) and 12/15-LOX pathways [111]. In turn, this causes the accumulation of a wide range of lipid mediators, including prostanoids, thromboxanes, lipoxins, and leukotrienes [112].

Early studies showed cPLA-2’s involvement in both initial and secondary injury phases in cerebral ischemic stroke, mediating oxidative and inflammatory responses [113,114]. Despite its established role in neurodegeneration and brain trauma, direct links between cPLA-2 and ferroptosis remain less explored. Beyond GSH systems, NADPH also influences ferroptosis; cPLA-2 interacts with NADPH oxidase subunits, promoting superoxide production in microglia [115,116,117]. However, the relative enzymatic activity of iPLA-2 in the brain dramatically exceeds that of cPLA-2 [118]. This discrepancy may be attributed to either the compensatory response of isoenzymes frequently observed in knockout mouse models or the complex species differences in PLA2 isotypes between rodents and primates. Evidence indicates the substantial presence of iPLA2 in cytosolic and nuclear fractions, as well as in mitochondrial and endoplasmic reticulum membranes, where it regulates oxPE-induced ferroptosis.

In contrast to pro-inflammatory cPLA-2, iPLA-2’s role in neuronal ferroptosis is more controversial, as it acts both as a sword and a shield. On the one hand, studies with rat fibroblasts unveiled the principal involvement of iPLA-2 but not c-PLA2 in the inflammatory regulation of sPLA-2 [119]. Prdx-6-iPLA-2 activity can promote astroglial and microglial activation, leading to the release of potent pro-inflammatory cytokines such as interleukin-1β (IL-1β) after CIR [120]. These studies exclusively point toward the role of iPLA-2 as a pro-inflammatory stimulus. On the other hand, inducible nitric oxide synthase (iNOS) can endow glia with anti-ferroptosis properties. iPLA-2 increases iNOS and cyclooxygenase-2 (COX-2) production in astrocytes and M1 microglia more than in M2 microglia, suggesting that the NO donor-induced nitration of eicosapentaenoic acid intermediates and truncated oxPE represents a novel redox mechanism for countering ferroptosis under pro-inflammatory conditions [120,121,122]. Neurons exhibit greater sensitivity to ferroptosis induced by RSL3 when genetic factors or chemical interventions lead to the loss or deficiency of iPLA-2, suggesting that iPLA-2 can inhibit ferroptosis to some extent. Meanwhile, the age-dependent decline in mRNA levels of iPLA-2 but not cPLA-2 in the rodent hippocampus partially explains why iPLA-2 is highly correlated with hippocampal health and cognitive functions [123]. iPLA-2 enhances lipid anti-peroxidation by either reducing negative trade-offs or providing positive synergies with the GPX4-dependent or -independent pathways, serving as an additional checkpoint to prevent unnecessary or excessive ferroptosis. Indeed, the role of the iPLA-2-related pathway may become essential when antioxidant thiol defenses collapse.

## 6. How the Nrf-2/ARE Pathway Inhibits Neuronal Ferroptosis in the Ischemic Brain: “Phospholipid Friends” and More

Nuclear factor erythroid 2-like 2 (Nrf-2) is a central transcriptional regulatory factor of endogenous antioxidant defense molecules for coping with oxidative stress and xenobiotic electrophiles. It functions by translocating to the nucleus and binding to antioxidant response elements (AREs) in gene promoters, including those of heme oxygenase-1 (HO-1), quinone oxidoreductase-1 (Nqo-1), and other phase II antioxidant enzymes. Melatonin activates cortical astrocytes through the PKCα/Nrf2/ARE signaling pathway, protecting against hemin-induced neurotoxicity and resisting oxidative stress in a mouse model of intracerebral hemorrhage (ICH) [124].

As an essential element of the cascade regulating phase II proteins, Nrf-2 gene deletion results in murine having low basal and mostly uninducible levels of these proteins, making them much more susceptible to neurotoxicity caused by hydrogen peroxide, permanent middle cerebral artery occlusion (p-MCAO), and cerebral edema due to scedosporiosis [125,126,127,128]. Nrf-2 targets AREs that play a crucial role in preventing lipid peroxidation. One key player, HO-1, acts as a rate-limiting enzyme in heme degradation, producing carbon monoxide, iron, and biliverdin. This positions Nrf-2 at the intersection of redox regulation and intermediary metabolism, highlighting its significance in cellular protection [129,130,131]. Under stable conditions, the Neh-2 and Neh-6 domains of Nrf-2 enable its interaction with partners like Kelch-like ECH-associated protein-1 (Keap1) and β-transducin repeats-containing proteins, leading to its incorporation into a Cullin-3 (Cul3) E3 ubiquitin ligase complex for proteasomal degradation, maintaining homeostasis. Nrf-2’s rapid turnover makes it one of the most transient proteins under normal circumstances. However, oxidative stress from events like stroke can disrupt this balance, causing electrophilic attacks on key reactive cysteine residues of Keap1 (C151, C273, and C288) [132]. The offensive launched by xenobiotic electrophiles inactivates Keap-1 so that Nrf-2 can be dislodged from the ubiquitin ligase complex to escape degradation [133]. Once Nrf-2 translocates from the cytoplasm to the nucleus, it forms a heterodimer with a small Maf transcription factor (sMaf) and then binds to cis-acting AREs to initiate the transcription of downstream target genes [134,135]. Therefore, Keap-1 negatively regulates the generation and translocation of Nrf-2, functioning as a redox sensor.

The transcription of Peroxiredoxin 6 (Prdx-6) activated by Nrf-2 enhances its non-selenium peroxidase properties and iPLA-2 activities, providing protection against ferroptosis [136]. This dual resistance is due to Prdx-6’s unique conformation, featuring a conserved cysteine residue and lacking a COOH-terminal, distinguishing it from other Prdx family members. Notably, Prdx-6 accumulates in astrocytes after hypoxia and redistributes in astrocytes and axons after reperfusion, indicating Nrf-2’s role in regulating Prdx-6 to combat oxidative stress [137]. Other key downstream targets of Nrf-2 in ferroptosis include Aldo-Keto Reductases 1B1 (AKR1B1) and 1B10 (AKR1B10), which are NADP(H)-dependent enzymes that metabolize aldehydes/ketones into low-toxicity alcohols, essential for detoxification in the brain and other tissues [138,139]. Neurogenic aldehydes and ketones, products of ω-6 PUFAs, contribute significantly to oxidative injury, with 4-hydroxy-2-nonenal (4-HNE) being the most abundant. For instance, 4-HNE readily forms toxic conjugates with glutathione, impacting neuronal glucose and glutamate transporters as well as Na+, K+-ATPase [140]. Additionally, 4-HNE protein adducts are used as markers for various types of brain damage and ferroptosis. Recent studies have indicated that the reduction of HNE by AKR1B1 converts the glutathione conjugate into a less toxic metabolite, 1,4-dihydroxynonene (DHN), suggesting that AKR1B1-catalyzed reduction is involved in the polyol pathway of intracellular signaling. Thus, they may be critical regulators of lipid peroxidation and ferroptosis. There is evidence linking cPLA-2 with AKR1B1, indicating that the 4-HNE arises from AAs generated by the extra cPLA-2. In turn, 4-HNE shows suppressing effects on the cPLA-2, but upregulates the Nrf-2 pathways in the BV-2 microglial cells challenged with lipopolysaccharide (LPS). The interplay between Peroxiredoxin 6 and Aldo-Keto Reductases in ferroptosis underscores critical neuroprotective mechanisms, revealing how Nrf-2-mediated pathways enhance neuronal resilience against oxidative stress.

**Figure 3:**
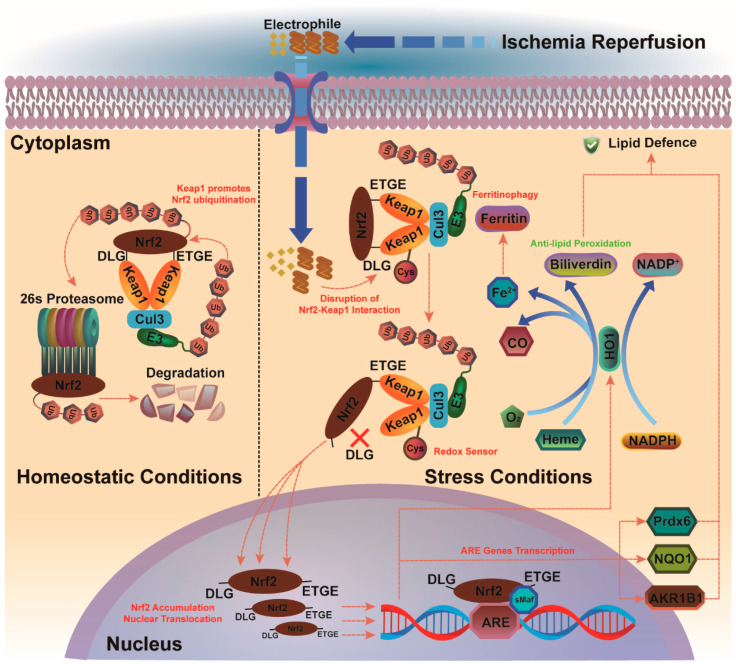
Mechanistic diagram of the Nrf-2/AREs signaling pathway. The first step is that the activation of the Nrf-2/AREs signaling pathway is initially attacked by the electrophile during ischemia and reperfusion. The cys assembles on Keap-1 as the redox sensor induces a conformational change that facilitates enhanced separation from Nrf-2 as ischemia and reperfusion upset the neuronal homeostatic balance. This partition stimulates the translocation of Nrf-2 from the cytoplasm into the nucleus, binding to sMaf and regulating the transcription of HO-1, Prdx-6, NQO-1, and AKR1B1, among others.

Nrf-2 could probably act as an upstream component of 4-HNE signaling, and 4-HNE also generates local negative feedback toward Nrf-2 through cPLA-2. Studies have taken it for granted that Nrf-2 is pivotal for regulating ferroptosis, especially during increased oxidative or metabolic stress. In brief, Nrf-2 mediates the crosstalk between ferroptotic lipid metabolism, Prdx-6, and antioxidant defense mechanisms in the hypoxic–ischemic brain, highlighting the interconnection between lipid defense against oxidative stress and ferroptosis (summarized in Figure 3).

## 7. Deuterated PUFAs: An Iconoclastic Way to Battle Ferroptosis

Deuterated PUFAs, substituted with the heavy hydrogen isotope deuterium at the site of peroxidation (D-PUFAs), such as with ^2^H by the dihydro–deuterium substitution reaction, have been shown to be strongly peroxide-resistant and can inhibit ferroptosis-related neurological disorders based on the primary kinetic isotope effect (KIE), though they are not nearly as potent as ferrostatin-1 and liproxstatin-1 [86,141,142]. Indeed, such modification has been shown to have robust utility toward mitigating autoxidation of the peroxidation products like 4-HNE and malondialdehyde (MDA) that can react with membrane components and nucleic acids to cause oxidative lesions [143].

Kinetic isotope effect (KIE) values peak at 80–100 when isotopically heavier hydrogen tunneling is leveraged as a prerequisite for intervention in the enzymatic reaction mechanism [144]. However, a limitation arises: only when the membrane bilayer incorporates D-PUFAs at a threshold concentration of approximately 20% can it effectively resist LPO. This is because hydrogen abstraction, compared to deuterium abstraction, is inherently more feasible [145]. Therefore, hydrogen abstraction by an oxidizer catalyzes the rate-limiting step in the synthesis of ROS, providing a clear illustration of the isotope effect (IE) and offering a novel perspective for assessing ferroptotic intensity in particular [146]. The resulting secondary by-products of LPO include endoperoxide and hydroperoxide intermediates, such as F2-isoprostanes (F2-IsoPs) and prostaglandin F2α (PGF2α). These compounds exhibit significant chemical stability, making them valuable indicators for LPO assessment. Elharram et al. found that supplementation with D-PUFAs restores normal cortical and hippocampal levels of F2-IsoPs and PGF2α in aldehyde dehydrogenase 2 (ALDH2)-deficient mice, which are unable to degrade toxic reactive carbonyl compounds derived from PUFAs. This indicates that D-PUFAs have a beneficial effect in preventing cognitive disturbances by alleviating LPO damage [147].

In addition, dietary supplementation with D-PUFAs reduces PUFA autoxidation, which is conducive to the protective response through increasing the level of mitochondrial HSP60 within the nigrostriatal dopaminergic pathway, as demonstrated in a 1-methyl-4-phenyl-1,2,3,6-tetrahydropyridine (MPTP) mouse model of Parkinson’s disease [142]. D-PUFAs, with their high affinity, exhibit excellent biological compatibility and facilitate effective pharmaceutical uptake and incorporation [148]. Additionally, the presence of dedicated transport mechanisms allows D-PUFAs to readily penetrate the BBB and enhance brain phospholipid metabolism [148,149]. Since isotope-reinforced PUFAs demonstrate efficacy in ameliorating LPO, they may have potential pharmacological applications for neurological diseases where LPO plays a significant detrimental role. However, a significant concern is that excessive use of D-PUFAs as novel therapeutic agents may exacerbate LOX-mediated enzymatic reactions due to the amplification of the KIE [150]. Therefore, determining the appropriate dosage of D-PUFAs is crucial to ensure compatibility with enzymatic transformations.

## 8. Matryoshka Doll: Subcellular Organelles in Ferroptosis

The plasma membrane and subcellular organelles are the common targets of lipid peroxidation, especially in different types of brain insult. The oxygen uptake and utilization of mitochondria account for 90% of the organism’s function, and oxygen consumption is counterbalanced by the production of ROS. Mitochondria, microsomes, endoplasmic reticulum, and other membrane-rich tissues are particularly abundant in polyunsaturated fatty acids (PUFAs), which are crucial for membrane functionality [151]. The solubility of oxygen in the non-polar lipid bilayer matrix is 7-8 times greater than in the polar matrix of cellular membranes, facilitated by high oxygen partial pressure following brain injury. Transition metal complexes, such as those in hemoglobin, can traverse the neurovascular endothelium and dissolve in the neuronal non-polar matrix, leading to an inevitable lipid oxidation crisis. However, cellular membranes enriched with docosahexaenoic acid (DHA) can degrade by up to 30% to reduce oxidation, which may be considered a compensatory neuronal protection mechanism, closely aligned with ischemic neuronal damage in rodents [152]. This accumulation of lipid peroxides further underscores a ferroptosis-vulnerable neuronal state.

This is a crucial finding in terms of the brain since it is the body’s primary consumer of oxygen and the first organ to suffer from oxygen deprivation. Consequently, it contains a high density of mitochondria to ramp up energy metabolism [153]. Despite its ambiguous role in ferroptosis, the mitochondrial membrane is a key target of oxidative stress during cerebral stroke, housing numerous membrane-bound redox carriers, such as iron-sulfur centers and quinones. Electron transfer occurs through the reoxidation of reductive coenzymes (quinones) along the electron transport chain (ETC) [154]. Hydrogen carried by the coenzyme is dehydrogenated into protons and free electrons, which are then transferred to molecular oxygen, forming ionic oxygen. These electrons couple with protons to generate ATP, thus maintaining mitochondrial energy metabolism stability within a normal cellular environment. However, iron overload in mitochondria can cause one-electron oxidation between redox carriers, resulting in the leakage of single electrons from the mitochondrial ETC substrate, leading to the production of highly reactive oxygen-derived radicals that damage the mitochondrial membrane [155,156]. In other words, the mitochondrial ETC does not always function as a reliable, insulated conductor. Instead, it can leak electrons both at the substrate end (ubiquinone) and the oxygen end (cytochrome C) when mitochondrial homeostasis is disrupted. The free electrons from the mitochondrial ETC are not involved in regulating the energy metabolism process of synthesizing ATP, but participate in the free radical metabolism process to irreversibly weaken mitochondrial membrane barrier function [156]. Consequently, neurons in an oxygen-deprived state often exhibit a highly reduced mitochondrial ETC, with abundant unpaired electrons prone to leakage. Meanwhile, blood flow and oxygen reperfusion at this inappropriate time fuel the possibility that the explosive leakage of electrons from the mitochondrial ETC will output a large number of oxygen free radicals to amplify the injury. The TCA cycle is another crucial mitochondrial metabolic process that enhances mitochondrial respiration and ROS production, particularly when supplemented with anaplerotic metabolites like glutamine [157]. During the post-reperfusion phase, restoration of oxygen and energy supply triggers the opening of the mitochondrial permeability transition pore (mPTP) and subsequent autophagy. This response is associated with mitochondrial fragmentation, cristae enlargement, and hyperpolarization of the mitochondrial membrane potential (MMP) [158,159].

Although the contributions of mitophagy in ischemic stroke are controversial, most expect that selectively degrading and eliminating abnormal or superfluous mitochondria can maintain brain energy metabolism [160]. But some earlier studies have reported that excessive stress-induced mitophagy can cause long-term cerebral problems, including focal or global cerebral ischemia or hypoxia–ischemia in various animal models [161,162,163]. Nevertheless, there are methods to quantitatively assess mitophagy and mitochondrial quality control, which can clarify the dual role of mitochondria—specifically, how the degradation of misfolded proteins can promote either protective or detrimental outcomes. The ubiquitin–proteasome system (UPS)’s machinery and mitophagy serve as complementary protective systems against certain cerebral ischemic processes, regulated by PINK-1, Parkin, NIX (BNIP3-like protein X), FUNDC-1, Bnip-3, and other related modulators [164,165].

PINK-1, a serine/threonine kinase with a C-terminal kinase domain and a mitochondrial targeting sequence at the N-terminus, facilitates its shuttling between the cytosol and mitochondria [166]. While PINK-1 accumulates on dysfunctional mitochondria and in the cytoplasm, it collaborates with Parkin in the same pathway to mitigate mitochondrial damage in Drosophila [167,168]. This mitochondrial localization supports the prior view that mitochondrial defects are involved in the pathophysiology of stroke or head injury [169]. PINK-1 artificially generates phospho-ubiquitin, prompting Parkin membrane translocation to mitochondria. The crosstalk between PINK-1 and Parkin becomes the minimal machinery for recruiting canonical autophagy players to launch a signaling cascade that activates mitophagy, but this remains controversial.

Alongside the essential PINK-1 activity described above, researchers have investigated if there are other alternative substrates with which PINK-1 degrades abnormal mitochondria. Richard J. Youle’s successful research has made a breakthrough in demonstrating that PINK-1 is not merely faithful in binding to Parkin. It initiates mitophagy via recruiting phospho-ubiquitin-mediated optineurin (OPTN), coiled-coil domain 2 (CALCOCO2), etc., to take its place, which ultimately results in the steady-state turnover of mitochondria [170]. Parkin is dedicated to expanding the mitophagy signal that promotes PINK-1 to generate more phospho-ubiquitin; thus, Parkin plays a limited but non-essential role in the mitophagy process. In addition to the canonical mitophagy pathway, under hypoxic conditions induced by stroke, the reduction of hydroxylated hypoxia-inducible factor (HIF) accelerates HIF accumulation, enhancing mitophagy sensitivity by activating FUNDC-1, Bnip-3, and NIX [171,172]. FUNDC-1 comprises a conserved LC3-interacting region (LIR), which directly connects with LC-3 or ATG-8 to promote the subsequent engulfment of mitochondria by autophagosomes. Under hypoxic conditions, FUNDC-1 can be dephosphorylated at serine 13 (Ser-13) by PGAM5 phosphatase through direct interaction, thereby enhancing the binding between LIR and LC-3. For example, research on CIR has shown that hypoxic preconditioning activates FUNDC1-dependent mitophagy to help mitigate CIR damage [173,174,175].

**Figure 4:**
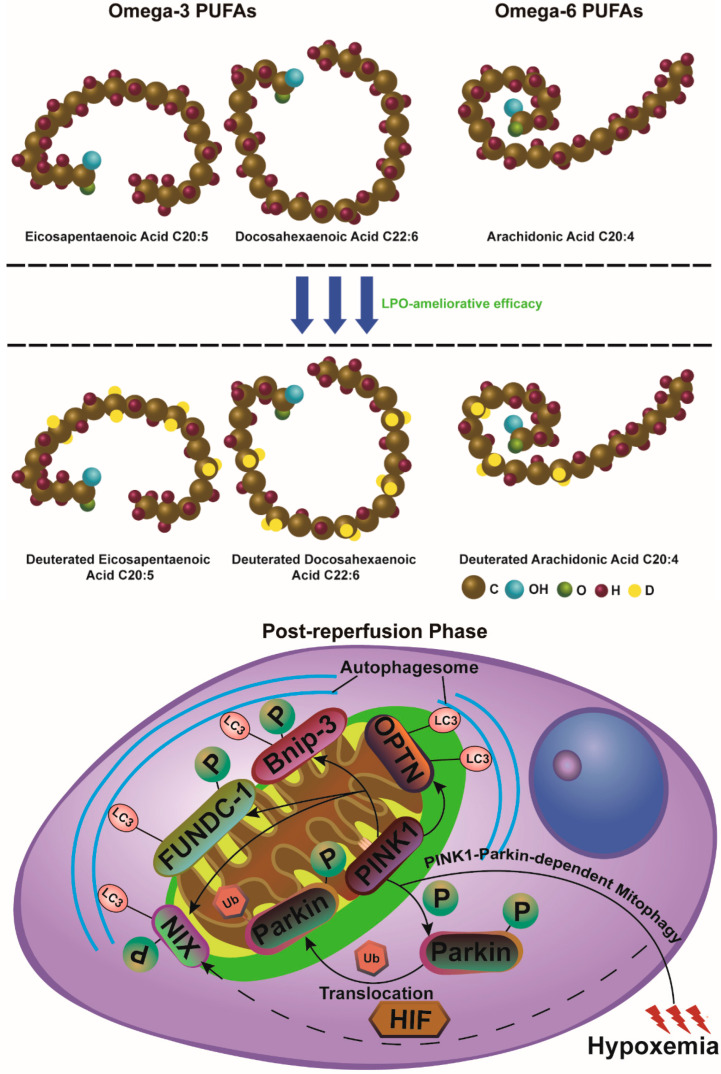
Three-dimensional representations of omega-3 and omega-6 PUFAs that are substituted with the heavy hydrogen isotope deuterium at the site of peroxidation in their spatially occupied conformations; Schematic representation of the cellular response in the PINK1–Parkin-dependent mitochondrial pathway under hypoxemic conditions. During the post-reperfusion phase, Parkin undergoes phosphorylation at various sites and actively participates in autophagosome formation.

Researchers believe that mitophagy genes exhibit a form of “friendship”, with members sharing the same LC3-interacting region (LIR) motif. Bnip-3 is specifically expressed on the mitochondrial outer membrane in the neurons of MCAO-treated mice. On this membrane, Bnip-3 instigates a mass recruitment of phagophores, precursors to autophagosomes, to mitochondria, by binding with Atg-8 family proteins under different hypoxic conditions, directly regulating the mitochondrial quality surveillance system and aiding PINK-1 in mitophagy induction [176,177,178,179]. Although both FUNDC-1 and Bnip-3 are involved in mitophagy induction, FUNDC-1 does not appear to influence neuronal mitophagy as extensively as Bnip-3, which contributes to the sequestration of mitochondria into autophagosomes, thereby triggering delayed neuronal death in stroke [180]. This notable difference in the regulation of neuronal mitophagy suggests that Bnip-3 may induce excessive mitophagy, leading to pronounced neuronal toxicity, while FUNDC-1 likely functions in a more coordinated manner, controlling baseline levels of mitophagy under hypoxic conditions, as previously suggested. Consequently, the role of Bnip-3 may have been underestimated in pathological conditions such as cerebral ischemia. Bearing significant amino acid homology to Bnip-3, NIX seems to wield a firmer hand in programmed mitochondrial elimination by regulating MMP, likely functioning as an alternative signaling pathway to the TCA cycle and ETC-driven ferroptosis [181,182]. Not coincidentally, NIX has been proven by clinical work and animal experiments to be a sensitive biomarker of ischemic injury before changes in histological characteristics can be detected [183,184]. Furthermore, significantly enhanced NIX protein levels and mitochondrial translocation are observed in rats subjected to MCAO after 6 h of reperfusion. The sensitive variation of NIX during the early stage of the CIR model and during neuronal lethality has indicated that it may serve as a potential target for extending the treatment time window, combined with thrombolytic therapy [184] (summarized in Figure 4).

Choi et al. have confirmed that Nrf-2 and the downstream targeted gene HO-1, as the ferroptotic signature, can stabilize HIF through rapid proteasomal degradation. This stabilization not only activates NIX in the early stages of ischemia but also promotes the production of vascular endothelial growth factor (VEGF), which is involved in angiogenesis during a later phase of ischemia [185,186]. Despite its continuous increase in the ischemic penumbra, the expression of HIF protein progressively decreases with prolonged early reperfusion [186]. The upstream switch in HIF expression also involves MEK/ERK pathways that are mainly responsible for the activation of translational machinery to inhibit the proteasomal degradation of its interacting proteins [187], while activation of the MEK/ERK pathway is imperative for Erastin-induced ferroptosis [188]. These processes are helpful in understanding how the neurons know the ferroptotic cues and hint at the idea that HIF can be a potential upstream responder for ferroptosis. Additionally, HO-1 stabilizes HIF by donating Fe^2+^ to proline hydroxylase (PHD), which uses O2 as a co-substrate to add a hydroxyl group specifically within the HIF oxygen-dependent degradation domain (ODD) [189]. Thus, excess Fe^3+^ is generated through peroxide-dependent oxidation of Fe^2+^, and the anaerobic conditions induced by ischemic stroke impose a dual attack on the catalytic activity of PHD.

There seems to be a positive feedback circuit in the Fe^2+^/HO-1/HIF/NIX signaling axis. It suggests that, beyond being directly activated by ischemic strokes, HIF as a paracrine mode of action can be stabilized by HO-1 to disable NIX-induced mitophagy, which shields neurons from ferroptosis. On balance, mitophagy integrated with ferroptosis has become a promising line of inquiry among the multiple intertwined mechanisms of endogenous defenses against stroke insult in specific pathological stages. The variable we need to consider is the threshold at which mitophagy undertakes a protective role in stroke through mitochondrial quality control.

## 9. Potential Therapeutic Targets of CIR: Ferroptotic Inhibitors

Focal cerebral ischemic stroke is a consequence of an interrupted or severely reduced blood supply to the specific part of the brain, leading to selective and delayed death of certain neuronal populations. Clinically, intravenous thrombolysis (IVT) combined with alteplase or intra-arterial thrombectomy remains the most effective FDA-approved intervention for ischemic stroke [190]. Though other complementary treatment avenues generally relating to antiplatelet aggregation, nerve-nurturing medicine, and improvement of blood microcirculation for secondary stroke prevention have subsequently emerged, patients treated using these methods often suffer from a moderate recanalization rate, inevitable intracranial hemorrhage tendency, insensibility during the therapeutic time window, and delayed neurotoxicity [1]. The development of the IVT field seems to be at an inexplicable standstill. Why has progress been so slow? Currently, CIR remains a significant global health challenge. Researchers have recently explored new treatment strategies to wrestle with such therapeutic dilemmas.

Several notable molecular mechanisms are involved with CIR, encompassing various inflammatory cytokines, LPO, ER stress, and mitophagy, within the core of ischemic penumbra injuries, though many more mechanisms underlying CIR remain largely mysterious. LPO has been identified and its presence has been reported in the pathogeneses of a whole range of acute encephalopathy conditions, including intracerebral hemorrhage, traumatic brain injury (TBI), and CIR [2,191].

Of note, the discovery of ferroptosis is all about the serendipity of its inhibitor, deferoxamine (DFO). Dixon et al. used a small molecule cancer drug named Erastin to induce tumor cell death, and the process could not be reversed by the inhibitors of apoptosis and necrosis other than DFO, hence the name ferroptosis [158]. DFO can cross the blood–brain barrier to chelate iron ions that accumulate in nerve tissue after CIR, thereby exerting a neuroprotective effect against ferroptosis [192,193]. Preclinical studies by Selim et al. demonstrated that continuous infusion of DFO is generally safe in patients with intracerebral hemorrhage and improves cerebral functional recovery, though not as rapidly as expected [194]. Thereby, DFO offers potential clinical benefits. Fer-1 can scavenge ferrous iron derived from lipid hydrogen peroxide and alkoxy free radicals in ischemic rodent brains and neuron-like cells, while Fer-1 itself remains unaffected [2,195]. Similarly, the ferritin inhibitor liproxstatin-1 (Lip-1) achieves ferroptotic inhibition by functioning as a free radical-trapping antioxidant (RTA) in OLN-93 oligodendrocytic cells [196]. So, are there other mechanisms that are independent of GPX4 for inhibiting ferroptosis? Different from Fer-1 and Lip-1, Vitamin E directly devitalizes the activity of the LOX enzyme to space out the process of LPO and restore the loss of GPX4/glutathione, so that it shows inhibition of ischemia-induced neural ferroptosis. Its prime mechanism is the process whereby Vitamin E is converted to quinone metabolites after ring-opening polymerization and subsequently undergoes a rapid reduction into benzenediol, while benzenediol eventually acts on the ferrous ions in the active center of the LOXs with high lipophilic properties [197,198,199]. Despite being a natural RTA, Vitamin E is less potent than the aromatic compound liproxstatin-1 (Lip-1) identified from high-throughput screening libraries [200]. It is worth heeding that neuro-endogenous ferroptosis suppressor protein 1 (FSP-1) with canonical N-terminal myristoylation can also convert quinone into reduced panthenol, sharing an identical GPX4 compensation effect and an anti-ferroptosis effect with Vitamin E, but the use of specific drugs involving N-terminal myristoylated FSP-1 or N-myristoyl transferase (NMT) is yet to be confirmed [23,201,202]. So, even if the influx of free radicals after CIR may smash the GPX4 frontier, FSP-1 N-terminal myristoylation can rescue neurons from a life-threatening crisis. Another GPX4-independent inhibitor is dihydroorotate dehydrogenase (DHODH). It is specifically involved in regulating ferroptosis in mitochondria [203]. Interestingly, uridine supplementation in mitochondrial DHODH-knockout cell lines can salvage cells from ferroptosis, but not in mitochondrial GPX4-knockout cell lines, suggesting that DHODH can develop its anti-ferroptosis function by enhancing pyrimidine nucleotide synthesis and can act in parallel but not synchronously with the GPX4 pathway to inhibit ferroptosis in mitochondria [204]. As a critical regulator of firing rate set points in hippocampal networks, DHODH can regulate the metabolic homeostasis of hyperexcitable hippocampal circuits when calcium overloads after CIR [205]. We may infer that DHODH and mitochondrial GPX4 maintain two major defensive methods against the accumulation of LPO in neuronal mitochondria. When both mechanisms are compromised, extensive ferroptosis primarily driven by mitochondrial lipid peroxidation is triggered.

An instructive age-dependent target for inhibiting ferroptosis, Tau protein, was revealed by Bi et al., and it rose to fame in various neurodegenerative diseases like Alzheimer’s disease and Parkinson’s disease, with a destructive function of forming intracellular tangles after phosphorylated modification [206,207]. But Tau sometimes exhibits antagonistic pleiotropy in stroke. Its ablation or inhibition has a substantial neuroprotective effect against CIR injury in a young group, but this effect is lost with age-related neurotoxic iron accumulation in the pro-ferroptosis iron pool, which underscores Tau’s ability to facilitate the unburdening of excess irons in neurons to inhibit ischemic ferroptosis [43,207]. The pleiotropic activity of Tau can assist us in revisiting and refocusing the balance between the neuroprotective role of Tau and the ferroptosis-inhibiting role of Tau based on the age distribution difference. Therefore, the above will provide some constructive and deliberate insights to help design and synthesize a new typical inhibitor of GPX4-independent ferroptosis. Other ferroptosis-related inhibitors in CIR include arylthiazyne derivatives, polyphenols, and some Chinese herb extracts, for example, curcumin, baicalein, epigallocatechin gallate, etc. [208,209,210,211,212], most of which restrain neuronal ferroptosis by preventing iron accumulation, GPX4 inactivation, GSH depletion, and LPO. Moreover, electroacupuncture (EA) is a therapeutic technique that combines traditional acupuncture with modern electrical stimulation, which is believed to mitigate ferroptosis and reduce CIR injury by activating the Nrf2 pathway [213]. Some compounds like Resolvins do not directly counteract ferroptosis in CIR; instead, they indirectly limit the process by inhibiting pro-inflammatory cytokines, reducing neutrophil migration, and promoting inflammation resolution [214,215]. Altogether, ferroptosis has three relatively independent mechanisms in subcellular localization, specifically, GPX4 in cytoplasm and mitochondria, FSP-1 in the plasma membrane, and DHODH in the mitochondria (summarized in Table 1).

## 10. Conclusions and Perspective

As a newly discovered programmed cell death pathway, ferroptosis differs from apoptosis, necrosis, or autophagy in its morphological and biochemical characteristics. Ferroptosis-related proteins are involved in the anoxemic death of neurons via initiating LPO and interfering with iron metabolism. Ferroptosis is also duplicitous because the pre-activation of ferroptosis could moderately exert a neuroprotective effect in the early stage of cerebral ischemia. However, the more vital role of ferroptosis, together with other programmed cell death types, has a negative effect on severe hypoxia–ischemia or the hyperactivation of neurons. Thus, regulating the occurrence of neuronal ferroptosis to relieve the related oxidative stress damage requires comprehensive consideration of the influence of multiple factors. In addition, although studies have shown that ferroptosis incidence is highly correlated with LPO, only a small number of ferroptotic inhibitors target lipid-rich mitochondria and cytomembrane with weak specificity. In the traditional sense, selenocysteine active sites in GPX4 and FSP-1 play decisive roles in resisting ferroptosis. Frankly, it is more of a mastermind than a perpetrator, and it also mixes with other types of programmed cell death. Only by searching out the specifically ferroptotic executioners can we develop specific inhibitors to effectively switch off ferroptosis while isolating the interactive risks of potential extraneous pathways. So, the ferroptotic executioners probably confine themselves to LPO-related molecules that condemn neurons to death. Developing highly specific inhibitors of neuronal ferroptosis based on LPO-related targets will be considerably predictable and feasible, which is conducive to gaining insight into the exact role of ferroptosis in neurological disease models for researchers. We had better not turn a blind eye to the staggering incidence of ischemic stroke worldwide, with its high mobility and mortality. Exploiting the mechanisms of crosstalk between ferroptosis and ROS may shed light on finding the hammer of Thor against ischemic stroke for us human beings.

## Figures and Tables

**Table 1:** This table categorizes various ferroptotic inhibitors based on their specific mechanisms in the context of ferroptosis modulation.

Ferroptotic Inhibitors	Mechanisms	
Drug	Consequence	Reference
Deferoxamine (DFO)	Chelates iron ions	[158,192,193]
Fer-1	Scavenges ferrous irons	[2,195]
Liproxstatin-1 (Lip-1)	Acts as a free radical trapping antioxidant (RTA)	[196]
Vitamin E	Inhibits LOX enzyme activity and restores loss of GPX4/glutathione	[197,198,199,200]
FSP-1		[23,201,202]
Dihydroorotate dehydrogenase (DHODH)	Enhances pyrimidine nucleotide synthesis	[204,205]
Tau	Unburdens excess irons in neurons	[43,206,207]
Electroacupuncture (EA)	Activates the Nrf2 pathway	[213]
Others (e.g., curcumin, baicalein, epigallocatechin gallate)	Prevents iron accumulation	[208,209,210,211,212]

## Data Availability

Individual data can be found in the referenced manuscripts.
